# Iodine-Based Wound Dressing Versus Antibiotic Therapy for Postoperative Symptom Relief in Third Molar Surgery

**DOI:** 10.7759/cureus.101748

**Published:** 2026-01-17

**Authors:** Natalija Golubenko, Jana Olak, Tiia Tamme, Armand Sutt, Janne Tiigimäe-Saar

**Affiliations:** 1 Department of Oral Surgery, Tartu University Hospital, Tartu, EST; 2 Department of Pediatric Dentistry, Tartu University Hospital, Tartu, EST; 3 Institute of Dentistry, University of Tartu, Department of Maxillofacial Surgery, Tartu, EST; 4 Statistics, KPEV Statistics OÜ, Tartu, EST; 5 Department of Maxillofacial Surgery, Tartu University Hospital, Tartu, EST

**Keywords:** antibiotics, iodoform gauze, oral wound dressings, postoperative pain, third molar extraction, wound healing

## Abstract

Objectives: This prospective, non-randomized comparative clinical study aimed to explore postoperative symptom outcomes associated with iodine-based wound dressings following third molar extraction, compared with conventional primary wound closure and postoperative antibiotic therapy.

Materials and methods: This prospective, non-randomized comparative clinical study was conducted at the Department of Oral and Maxillofacial Surgery, Tartu University Hospital, between May 1, 2022, and December 31, 2023.A total of 68 patients undergoing third molar extraction were assigned to four groups based on postoperative management. Group A received antibiotic therapy, Group B received iodoform gauze, Group C underwent extraction of both upper and lower third molars with iodoform gauze applied to the lower site, and Group D served as a control group without preventive measures. Postoperative pain, swelling, chewing difficulty, and drowsiness were assessed using questionnaires and visual analog scales where applicable.

Results: Patients treated with iodoform gauze (Groups B and C) demonstrated postoperative outcomes comparable to those observed in the antibiotic group (Group A), with a non-significant trend toward shorter pain duration. Group C reported the most favorable subjective recovery outcomes. The control group experienced the longest duration of postoperative pain and chewing difficulties.

Conclusion: Local iodine-based wound management following third molar extraction is a comparable option to systemic antibiotic therapy.

## Introduction

The eruption status, anatomical position, and angulation of impacted third molars affect the associated symptoms, which may manifest as pericoronitis, pain, swelling, pathological changes of adjacent teeth and bone, and the development of odontogenic cysts or tumors[1]. Common postoperative complications following third molar removal include pain, facial swelling, trismus, alveolar osteitis (dry socket), temporary or permanent neurosensory disturbances of the inferior alveolar nerve, and disruption of psychosocial well-being[2].

Although antibiotics have been used to prevent postoperative infections [3], current clinical guidelines from the American Dental Association (ADA), American Heart Association (AHA), and National Institute for Health and Care Excellence (NICE) discourage routine prophylactic use in healthy patients undergoing third molar extraction [4-6].

Due to increasing antimicrobial resistance, local antiseptics are being explored as alternative infection control strategies, offering broad-spectrum antimicrobial activity with a lower risk of resistance development [7]. Preventive pharmacological measures typically include analgesics and corticosteroids [2], whereas unnecessary antibiotic use increases the risk of resistance and adverse effects [3,4].

In cases of alveolitis, common oral and opportunistic pathogens include facultative streptococci, anaerobic Gram-negative bacteria, and Candida species [6-12]. Although preoperative antibiotic administration can reduce the risk of alveolitis, key factors for preventing postoperative complications include proper preoperative disinfection, sterile instrumentation and irrigation during surgery, good postoperative oral hygiene, and the use of local antiseptics [3].

Among iodophores, povidone-iodine and iodoform are the most widely used. Iodoform (triiodomethane) is a yellow crystalline organic halogen compound with antiseptic properties [8,10]. Povidone-iodine is a complex of iodine and povidone that exerts microbicidal activity [7], and iodine exhibits broad-spectrum antimicrobial activity against bacteria, fungi, viruses, and yeasts [12]. Iodoform gauze is considered suitable for the treatment of dry sockets and complicated wounds due to its rapid and effective pain relief, non-irritating nature, ease of absorption, antiseptic properties, and resistance to oral fluids[8,13].

Some in vitro studies have shown that iodine-based dressings can exert cytotoxic effects on fibroblasts and epithelial cells at specific concentrations [14]. However, these effects are context-specific and may not directly translate to clinical outcomes. Safer and regenerative alternatives, including platelet-rich fibrin (PRF) [15,16] and concentrated growth factors (CGFs) [17,18], have also been explored in earlier studies to enhance wound healing and tissue regeneration.

The aim of this prospective comparative study is to evaluate the clinical effectiveness of iodoform gauze drain and secondary wound healing compared with conventional primary closure and antibiotic therapy, focusing on postoperative clinical outcomes.

## Materials and methods

Study design and participiants

This prospective, non-randomized comparative clinical study was conducted at the Department of Oral and Maxillofacial Surgery, Tartu University Hospital, between May 1, 2022, and December 31, 2023.Patients aged 16 to 80 years with a partially or fully impacted lower third molar requiring extraction for medical reasons were eligible for inclusion.

Exclusion criteria included surgical procedures that significantly deviated from the standardized extraction protocol described in this study or required specialized surgical instruments, as well as the presence of significant physical or mental health conditions that could interfere with treatment or postoperative recovery. Patients with an American Society of Anesthesiologists (ASA) physical status ≥ III [19].

A total of 68 patients met the inclusion criteria and were enrolled in the study. Participants were assigned to one of four groups based on their informed preference for postoperative management, resulting in a non-randomized, preference-based allocation (Table 1).

**Table 1:** Study groups

Group	Group description
A	Patients who received antibiotic therapy
B	Patients who received an iodoform gauze drain
C	Patients who received an iodoform gauze drain and had both upper and lower third molars extracted on the same side
D	Control group without preventive measures

All participants were informed about all available postoperative management options, including systemic antibiotic therapy, local iodoform gauze drain placement, or no antibacterial prophylaxis. The potential benefits, limitations, and practical aspects of each approach were explained in detail. No predefined clinical indications mandated the use of one strategy over another, and the final decision was made collaboratively based on the patient’s informed preference. In cases involving simultaneous maxillary and mandibular third molar extraction (Group C), patients were additionally informed about the more extensive nature of the procedure and the potential implications for postoperative care.

Group A included patients who receivedpostoperative prophylactic antibiotictherapy after lower third molar extraction (n = 22). Group B included patients who received postoperative prophylactic iodoform gauze placement in the extraction site (n = 23). Group C comprised patients who underwent extraction of both maxillary and mandibular third molars on the same side, with a prophylactic iodoform gauze drain placed only in the lower extraction site (n = 11). No postoperative systemic antibiotics were administered in these groups. Group D served as the control group and included patients who did not receive any postoperative antibacterial prophylaxis (n = 12).

Preference-based group allocation was used to reflect real-world clinical decision-making; however, this approach introduces potential selection bias and confounding, particularly in Group C. These limitations should be considered when interpreting the results. Due to the nature of the interventions (systemic antibiotic therapy versus local wound dressing), blinding of patients and operators was not feasible.

Pre- and postoperative use of analgesics, antibiotics, and corticosteroids was recorded for all patients. In Group A, postoperative systemic antibiotic therapy consisted of amoxicillin with clavulanic acid (875 mg/125 mg) administered twice daily for a seven-day course. No patients in the antibiotic group had a documented allergy to penicillin or related antibiotics.

Impacted mandibular third molars were classified visually according to Pell and Gregory for descriptive purposes; these data were not analyzed in relation to postoperative outcomes. No formal preoperative periodontal measurements were performed. Intra- and postoperative complications were documented, including acute bleeding, root displacement, inferior alveolar nerve visualization, infection, alveolar osteitis, nerve injury, and mandibular fracture.

Surgical procedure

The duration of the surgical procedure was measured from the first incision to the final suture. All extractions were performed using a standardized trapezoidal flap design to ensure procedural consistency. For wound closure, resorbable sutures (Vicryl 5-0, ETHICON, Johnson & Johnson) were used.

**Figure 1:**
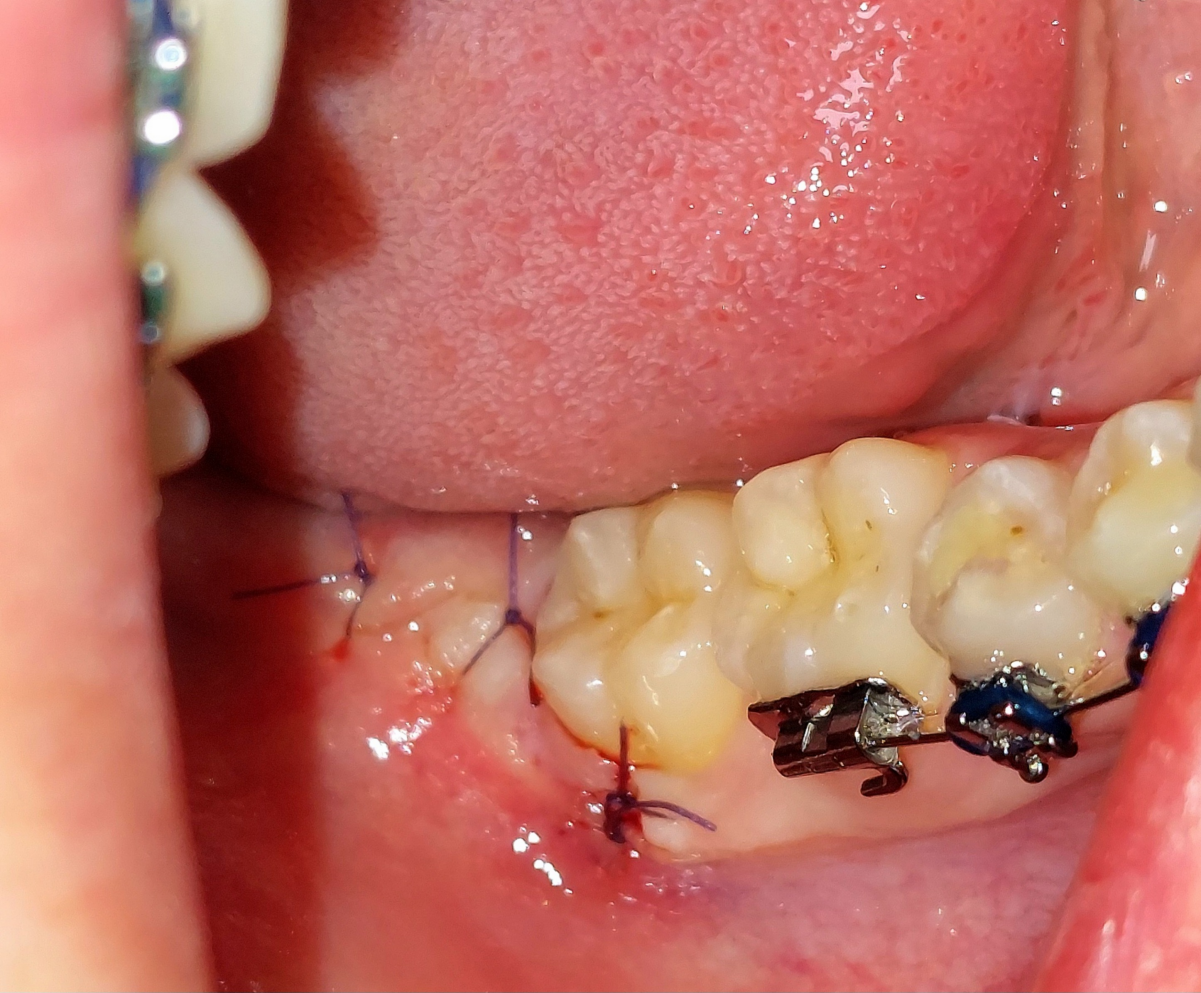
Extraction site of the wisdom tooth closed primarily with sutures. Photo taken by the author (private collection). Patient consent for publication of anonymised image was obtained.

**Figure 2:**
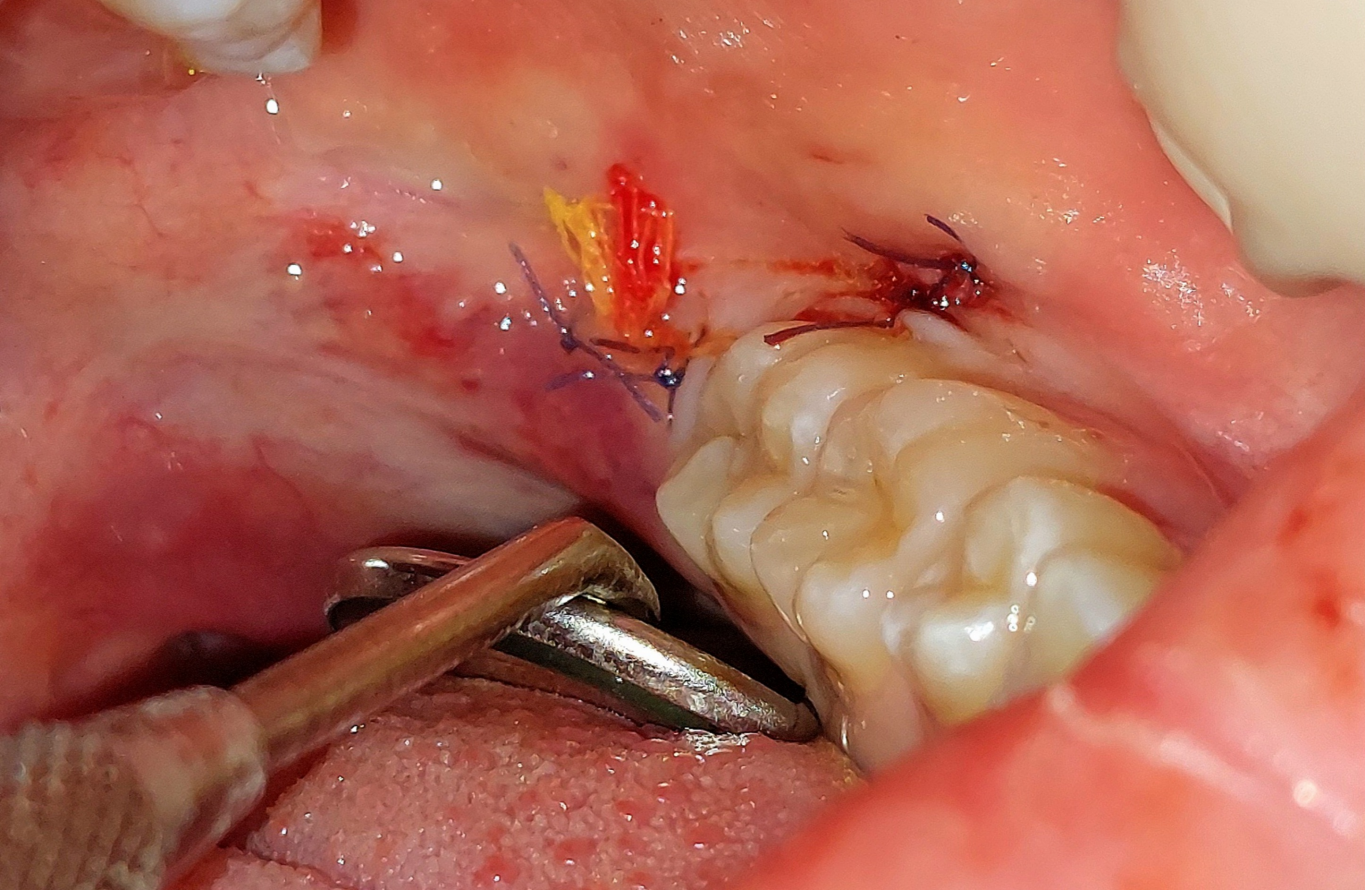
Extraction site closed secondarily with sutures and an iodoform gauze drain. Photo taken by the author (private collection). Patient consent for publication of anonymised image was obtained.

In Groups A and D, the extraction site was closed primarily (Figure1). In Groups B and C, a small opening was left after wound suturing to allow insertion of a 1.5-2 cm iodoform gauze drain (Figure2).

In Group C, extraction of the upper third molar was performed following removal of the lower third molar. Upper molar wounds were revised and either primarily closed with 5-0 Vicryl sutures or left to heal by secondary intention without placement of an iodoform gauze drain.

All procedures were performed by two experienced oral surgeons following an identical standardized protocol; no inter-operator deviations occurred.

Postoperative assessment

Postoperative outcomes were assessed two weeks after surgery using a standardized self-administered questionnaire (see Appendix). Pain intensity was recorded retrospectively at the two-week follow-up.The questionnaire evaluated pain intensity, facial swelling, trismus, social and occupational limitations, physical appearance, ability to eat and speak, dietary changes, sleep disturbances, oral health-related quality of life (OHRQoL), and overall postoperative discomfort. Responses were scored on a 4-point ordinal scale (0 = no, 1 = somewhat, 2 = significantly, 3 = yes).

The questionnaires used in our study were based on those published in the study by Dr. Tiigimäe-Saar, one of the co-authors the current manuscript [2]. The original questionnaires were modified according to the specific requirements of our study and analyzed with respect to the relevant outcome measures.

Pain intensity and sensory disturbances were additionally assessed using a Visual Analogue Scale (VAS) ranging from 0 to 100, where 0 indicated no pain or sensory disturbance and 100 indicated maximum pain or complete numbness. The OHIP-14 questionnaire and the Visual Analogue Scale (VAS) used for pain and sensory assessment are widely validated and non-licensed instruments, commonly applied in oral surgery research [2].

Patients were also asked to report sensory disturbances affecting the lower lip, chin, and tongue, as well as their willingness to undergo the procedure again or recommend it to relatives if clinically indicated.

All enrolled patients completed the two-week postoperative follow-up. No dropouts or losses to follow-up were recorded.

Preparation of iodoform gauze drains

**Figure 3:**
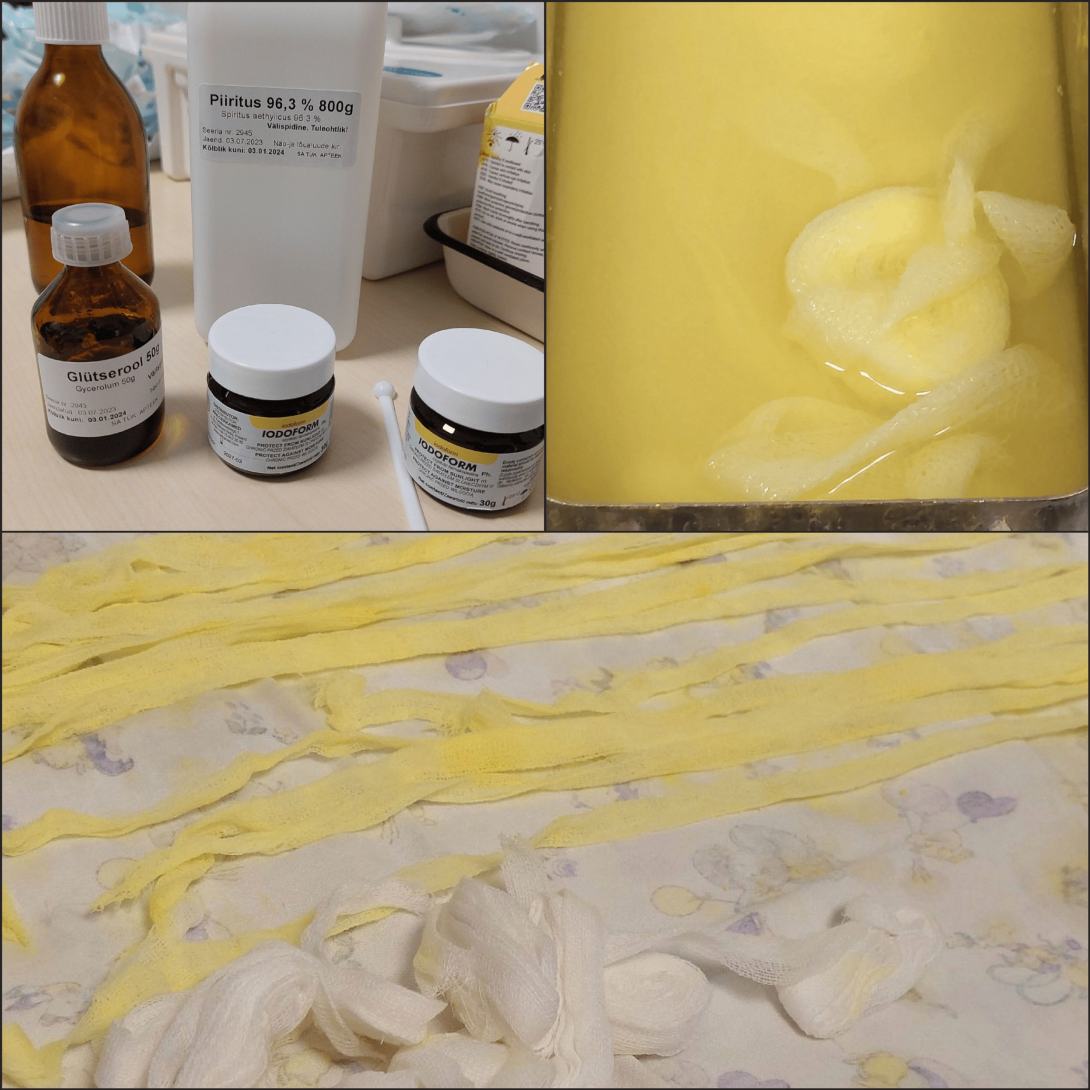
Preparation of the iodoform gauze tampon-drain. Photo taken by the author (private collection).

Iodoform gauze drains were prepared under sterile conditions following the protocol described by Sailer H.F. and Pajarola G.F. (1999)[20]. Sterile gauze tampons were soaked in a solution containing 75 ml of ether, 25 ml of glycerol, and 75 ml of 96.3% ethanol. After soaking, excess liquid was removed, and 20 g of iodoform powder was thoroughly mixed into the gauze. The tampons were then dried between sterile textile layers for approximately 24 hours (Figure3).

The finished gauze drains were not autoclaved or heated, to avoid degradation of iodoform, and were stored in sterilized containers until use.

Statistical analysis

Statistical analyses were performed using RStudio (Posit PBC, Massachusetts, USA).

Continuous variables, including postoperative pain duration, chewing difficulty, and drowsiness, were reported as means. Categorical variables, such as gender, extraction indication, and subjective recovery expectations, were reported as counts and percentages. Dry socket, infection, trismus, swelling, and nerve injury were monitored in all groups; no statistically significant differences were detected, and therefore these outcomes are not presented in detail.

Postoperative outcomes were analyzed descriptively and using pairwise comparisons between groups, focusing on pain duration, chewing difficulties, postoperative drowsiness, and subjective recovery assessments. For comparisons involving more than two groups, one-way analysis of variance (ANOVA) was used to assess overall group differences for continuous outcome variables. Where appropriate, exploratory linear regression models with treatment group as a categorical predictor were additionally fitted to estimate effect sizes and directionality of group differences.

Between-group comparisons for continuous variables were conducted using independent-samples t-tests (exploratory) and are reported descriptively. These analyses were considered exploratory and were not used as confirmatory evidence due to the increased risk of type I error associated with multiple testing. For categorical variables, the Chi-square test or Fisher’s exact test was applied as appropriate for small sample sizes.

A p-value < 0.05 was considered statistically significant. Given the exploratory nature of the study and non-randomized group allocation, findings were interpreted cautiously.

Ethical approval and informed consent

The study was approved by the Clinical Ethics Committee of the University of Tartu (Approval No. 362/T-2, issued on April 18, 2022, valid until December 31, 2029). All participants provided written informed consent prior to inclusion in the study, including consent for the use of anonymised images in this manuscript. The research was conducted in accordance with the principles of the Declaration of Helsinki.

## Results

A total of 68 patients were included and allocated to four groups based on patient preference: antibiotic therapy (A, n = 22), iodoform gauze drain (B, n = 23), dual-extraction with iodoform gauze drain (C, n = 11), and control (D, n = 12) (Table2). The mean age ranged from 20.09 years in group C to 27.04 years in group A. Acute indications for extraction were most frequent in group B (n=12) and absent in group D (Table3).

**Table 2:** Demographics of the study participants.

	A	B	C	D
Male	7	9	8	2
Female	15	14	3	10
Total	22	23	11	12
Group mean age	27.04	24.83	20.09	22.67

**Table 3:** Indication for extraction by group.

	A	B	C	D
Acute condition	4	12	1	0
Non-acute condition	18	11	10	12

Mean postoperative pain duration ranged from 4.0 days in group A to 6.75 days in group D, with an exploratory pairwise difference between groups A and D (p = 0.02) (Table 4). One-way ANOVA did not reveal a statistically significant overall group effect, F(3,64) = 2.38, p = 0.078, although a tendency toward longer pain duration in the control group was observed. Exploratory pairwise comparisons suggested a difference between groups A and D; however, these findings should be interpreted cautiously, as the omnibus test did not reach statistical significance.

**Table 4:** Postoperative pain duration (days) and pairwise p-values between groups. Overall group differences were assessed using one-way ANOVA. Pairwise t-tests are presented for exploratory purposes only. ANOVA (overall group comparison): F(3,64) = 2.38, p = 0.078

Pain (days)	A	B	C	D
A	4.00			
B	t(27) = -0.31, p = 0.38	4.36		
C	t(26) = -1.61, p = 0.06	t(19) = -1.26, p = 0.11	5.90	
D	t(28) = -2.08, p = 0.02	t(21) = -1.60, p=0.06	t(20) = -0.56, p=0.29	6.75

The duration of postoperative chewing difficulty was shortest in group C (1.80 days) and longest in group D (4.25 days); exploratory pairwise differences were observed between group C and groups A and D (Table 5). The overall group comparison using one-way ANOVA did not demonstrate a statistically significant difference, F(3,64) = 0.17, p = 0.91. While exploratory analyses indicated variability between treatment groups, no robust differences were confirmed after accounting for multiple group comparisons. These findings should be interpreted as hypothesis-generating only.

**Table 5:** Duration of chewing difficulties (days) across study groups. No statistically significant overall group effect was observed. ANOVA (overall group comparison): F(3,64) = 0.17, p = 0.91

Chewing Difficulty (days)	A	B	C	D
A	4.11			
B	t(27) = 0.50, p = 0.31	3.54		
C	t(26) = 1.97, p = 0.03	t(19) = 1.83, p=0.04	1.80	
D	t(28) = -0.12, p = 0.55	t(21) = -0.69, p=0.75	t(20) = -2.84, p=0.01	4.25

Postoperative drowsiness differed more clearly between groups C (2.00 days) and B (0.91 days). Statistically significant differences were observed between group C and groups A and B (p = 0.01 for both comparisons). One-way ANOVA demonstrated a statistically significant overall group effect, F(3,64) = 3.40, p = 0.023 (Table 6). Exploratory regression analysis suggested differences in drowsiness duration between treatment strategies. Given the non-randomized group allocation and differences in clinical presentation, this finding should be interpreted with caution.

**Table 6:** Duration of postoperative drowsiness (days) across study groups. Exploratory regression analysis indicated lower mean drowsiness duration in group C compared with group A. ANOVA (overall group comparison): F(3,64) = 3.40, p = 0.023

Drowsiness (days)	A	B	C	D
A	1.11			
B	t(27) = 0.55, p = 0.29	0.91		
C	t(26) = 2.68, p=0.01	t(19) = 2.43, p=0.01	2.00	
D	t(28) = -0.15, p=0.56	t(21) = -0.66, p=0.74	t(20) = -2.77, p=0.01	1.17

Subjective recovery outcomes are presented in Table 7. All patients in group C reported a better-than-expected postoperative course, whereas the majority of patients in groups A, B, and D also reported positive recovery experiences. Due to the descriptive nature of this outcome, no inferential statistical testing was applied.

**Table 7:** Subjective postoperative recovery expectations by group. Values in parentheses represent percentages of the total group.

Group	No (%)	Yes (%)
A	8 (44)	10 (56)
B	4 (36)	7 (64)
C	0 (0)	11 (100)
D	5 (42)	7 (58)

At the omnibus level, no statistically significant group differences were observed for pain duration, chewing difficulty, or patient-reported satisfaction. An a priori power calculation was not performed, as the study was designed as an exploratory, prospective, non-randomized clinical investigation with preference-based group allocation. Accordingly, results are presented descriptively and interpreted cautiously.

## Discussion

The present study indicates that postoperative outcomes following the use of an iodoform gauze drain with secondary wound healing were comparable to those observed with conventional primary closure and systemic antibiotic therapy.

Systemic antibiotics are commonly used to reduce the risk of postoperative infection following third molar extraction; however, several studies have questioned their routine prophylactic use in healthy patients. Sathish *et al*. [21] reported no significant difference between preoperative and postoperative antibiotic administration, while Strach-Jensen *et al*. [2] showed that amoxicillin and amoxicillin-clavulanic acid remain the most frequently prescribed agents. Sologova *et al*. [3] and Siddiqi *et al*. [22] demonstrated that only a small proportion of patients benefit from prophylactic antibiotics [23], supporting current international guidelines that emphasize antibiotic stewardship and restrict routine use to clearly defined high-risk cases [4-6].

Iodine-based dressings provide broad-spectrum antimicrobial activity and remain effective against a wide range of oral pathogens. Although in vitro studies have demonstrated potential cytotoxic effects of iodine on fibroblasts depending on concentration and exposure time [24], such effects were not assessed clinically in the present study, and no signs of iodine intolerance or adverse reactions were observed. Known contraindications to iodine use, including thyroid disease, pregnancy, and renal failure, should nevertheless be considered in clinical practice [7,8,12].

Previous studies have reported the beneficial effects of iodine-based wound management in oral surgery. Povidone-iodine irrigation has been associated with reduced postoperative swelling [25,26], and iodoform-containing dressings have demonstrated antibacterial efficacy and reduced early postoperative complications [10,25-27]. These findings are consistent with the comparable clinical outcomes observed in the present study.

In contrast to iodine-based dressings, regenerative approaches such as PRF and CGFs aim to enhance tissue regeneration rather than primarily provide antimicrobial effects. Systematic reviews suggest that PRF may reduce pain, swelling, and alveolar osteitis [28-30]; however, these modalities serve different biological purposes and should be viewed as complementary rather than directly interchangeable.

The interpretation of the present findings is limited by the non-randomized study design and the higher proportion of acute cases in the iodoform groups, which may have influenced baseline symptom burden and patient-reported outcomes. These limitations highlight the need for larger, randomized controlled trials to confirm the observed trends and to more precisely define the role of iodine-containing gauze drains in postoperative management following third molar extraction.

## Conclusions

Importantly, the use of an iodoform gauze drain with secondary wound healing was not associated with inferior postoperative outcomes when compared with conventional primary closure and systemic antibiotic therapy.

These results suggest that local iodine-based wound management may represent a feasible alternative to routine postoperative antibiotic use in selected patients undergoing third molar extraction. However, larger randomized controlled trials are required to confirm these findings and to define the role of iodine-containing dressings within evidence-based postoperative care protocols.

## Appendices

Study questionnaires

Questionnaire T0 (Preoperative)

Completed by the physician during medical history taking.

1. Patient Identification and Demographics Patient ID: __________ Age (years): _______ Sex: ☐ Male ☐ Female 2. Clinical Background and Preoperative Status Comorbidities: ☐ None ☐ Present (please specify): __________ Oral health status: ☐ Good (minimal previous dental treatment) ☐ Moderate (moderate dental treatment history) ☐ Poor (extensive dental treatment history) Preoperative radiographic examination: ☐ Periapical radiograph ☐ Orthopantomography (OPTG) ☐ Cone-beam computed tomography (CBCT) Impaction type of the mandibular third molar (Pell & Gregory classification):________ Preoperative symptoms: ☐ Pain ☐ Swelling ☐ Inflammation ☐ Dental caries ☐ Periodontal disease ☐ Other: __________ Preoperative pathology: ☐ None ☐ Cyst ☐ Benign tumor ☐ Other: __________ Indication for extraction: ☐ Acute ☐ Non-acute Preoperative anxiety related to the upcoming surgery (VAS 0-100): _______ (0 = no anxiety; 100 = highest imaginable anxiety) Preoperative analgesic medication (drug name): __________ Preoperative antibiotic medication (drug name): __________

Oral Health Impact Profile (OHIP-14)

Completed by the patient before surgery, referring to the previous six months.

Please circle how frequently you have experienced the following due to problems with your teeth, mouth, or dentures.

Have you had trouble pronouncing any words? 0 = Never 1 = Hardly ever 2 = Occasionally 3 = Fairly often 4 = Very often

Have you felt that your sense of taste has worsened? 0 = Never 1 = Hardly ever 2 = Occasionally 3 = Fairly often 4 = Very often

Have you experienced painful aching in your mouth? 0 = Never 1 = Hardly ever 2 = Occasionally 3 = Fairly often 4 = Very often

Have you found it uncomfortable to eat certain foods? 0 = Never 1 = Hardly ever 2 = Occasionally 3 = Fairly often 4 = Very often

Have you been self-conscious because of dental or oral problems? 0 = Never 1 = Hardly ever 2 = Occasionally 3 = Fairly often 4 = Very often

Have you felt tense because of dental or oral problems? 0 = Never 1 = Hardly ever 2 = Occasionally 3 = Fairly often 4 = Very often

Has your diet been unsatisfactory because of dental or oral problems? 0 = Never 1 = Hardly ever 2 = Occasionally 3 = Fairly often 4 = Very often

Have you had to interrupt meals because of dental or oral problems? 0 = Never 1 = Hardly ever 2 = Occasionally 3 = Fairly often 4 = Very often

Have you found it difficult to relax because of dental or oral problems? 0 = Never 1 = Hardly ever 2 = Occasionally 3 = Fairly often 4 = Very often

Have you been embarrassed because of dental or oral problems? 0 = Never 1 = Hardly ever 2 = Occasionally 3 = Fairly often 4 = Very often

Have you been irritable with other people because of dental or oral problems? 0 = Never 1 = Hardly ever 2 = Occasionally 3 = Fairly often 4 = Very often

Have you had difficulty performing your usual activities because of dental or oral problems? 0 = Never 1 = Hardly ever 2 = Occasionally 3 = Fairly often 4 = Very often

Have you felt that life in general was less satisfying because of dental or oral problems? 0 = Never 1 = Hardly ever 2 = Occasionally 3 = Fairly often 4 = Very often

Have you been totally unable to function because of dental or oral problems? 0 = Never 1 = Hardly ever 2 = Occasionally 3 = Fairly often 4 = Very often

Dental Anxiety (Modified Dental Anxiety Scale, MDAS)

Completed by the patient before surgery.

Please circle the response that best describes how you would feel in each situation. If you had a dental appointment tomorrow, how would you feel? = Not anxious 2 = Slightly anxious 3 = Moderately anxious 4 = Very anxious 5 = Extremely anxious

If you were sitting in the waiting room waiting for your dental appointment, how would you feel? = Not anxious 2 = Slightly anxious 3 = Moderately anxious 4 = Very anxious 5 = Extremely anxious

If you were about to have a tooth drilled, how would you feel? = Not anxious 2 = Slightly anxious 3 = Moderately anxious 4 = Very anxious 5 = Extremely anxious

If you were about to have your teeth scaled and polished, how would you feel? = Not anxious 2 = Slightly anxious 3 = Moderately anxious 4 = Very anxious 5 = Extremely anxious

If you were about to receive a local anesthetic injection in the gum, how would you feel? = Not anxious 2 = Slightly anxious 3 = Moderately anxious 4 = Very anxious 5 = Extremely anxious

Scoring: The total MDAS score is calculated by summing the responses to all five questions (range 5-25). A score ≥19 indicates high dental anxiety.

Questionnaire T1 (Postoperative)

Completed by the physician after surgery.

Patient ID:

1. Surgical Data Teeth extracted during surgery: ☐ Mandibular third molar only ☐ Ipsilateral maxillary and mandibular third molars Duration of surgery (minutes; from first incision to final suture): _______ Intraoperative complications: ☐ None ☐ Bleeding ☐ Root displacement into surrounding tissues ☐ Inferior alveolar nerve exposure ☐ Nerve injury ☐ Mandibular fracture ☐ Other_______

2. Postoperative Management Postoperative prophylactic approach: ☐ Systemic antibiotic therapy (drug name): __________ ☐ Iodoform gauze drain ☐ None Postoperative analgesic medication (drug name): __________ Preoperative and/or postoperative corticosteroid use: ☐ No ☐ Yes (drug name): __________

Continuation of the questionnaire at 2 weeks postoperatively. 3. Patient-Reported Outcomes at 2 Weeks Completed by the patient after surgery.

Visual Analogue Scale (VAS) Please rate the maximum intensity of pain experienced after wisdom tooth extraction on a scale from 0 to 100. 0 = no pain 100 = worst pain imaginable 0 10 20 30 40 50 60 70 80 90 100.

Please indicate whether you experienced any of the following within two weeks after surgery: ☐ Alveolar osteitis (dry socket) ☐ Postoperative infection ☐ Trismus (limited mouth opening) ☐ Clinically relevant facial or oral swelling ☐ Sensory nerve injury (persistent numbness or altered sensation) ☐ None of the above

4. Oral Health-Related Quality of Life (OHRQoL) Please answer the following questions by selecting the appropriate option (Yes/No) and, if applicable, indicating the number of days.

4.1. Social Activity Limitation Q1. Have you continued participating in your usual daily activities after surgery? ☐ Yes ☐ No Q2. On how many days after surgery did you refrain from your usual activities? Days: ___

Q3. Have you continued practicing your favorite sport or leisure activity after surgery? ☐ Yes ☐ No Q4. If yes, on how many days after surgery did you refrain from sports or leisure activities? Days: ___ Q5. Did you experience postoperative pain? ☐ Yes ☐ No Q6. If yes, on how many days after surgery did you experience pain? Days: ___ Q7. Did you experience more postoperative pain than expected? ☐ Yes ☐ No

Q8. Did you develop postoperative swelling? ☐ Yes ☐ No Q9. If yes, on how many days did you experience postoperative swelling? Days: ___ Q10. If yes, was the postoperative swelling greater than expected? ☐ Yes ☐ No

Q11. Did you notice changes in your appearance? ☐ Yes ☐ No Q12. If yes, on how many days did you perceive changes in your appearance? Days:___ Q13. Did you experience mood changes? ☐ Yes ☐ No Q14. Did you experience discomfort? ☐ Yes ☐ No Q15. Did you notice numbness of the lower lip after surgery? ☐ Yes ☐ No Q16. How would you rate the change in lower lip sensation? ___ (0-100; 0 = complete numbness; 100 = normal sensation)

Q17. Did you notice numbness of the chin after surgery? ☐ Yes ☐ No Q18. How would you rate the change in chin sensation? ___ (0-100; 0 = complete numbness; 100 = normal sensation)

Q19. Did you notice numbness of the tongue after surgery? ☐ Yes ☐ No Q20. How would you rate the change in tongue sensation? ___ (0-100; 0 = complete numbness; 100 = normal sensation)

4.2. Work-Related Limitation Q21. Did you request sick leave or interrupt your work activities? ☐ Yes ☐ No Q22. For how many days were you on sick leave after surgery? Days: ___ Q23. Did the surgery affect your ability to perform your work? ☐ Yes ☐ No

4.3. Eating and Diet Q26. Did you continue consuming your usual diet? ☐ Not at all ☐ To some extent ☐ Significantly ☐ Yes Q27. On how many days after surgery did you experience eating difficulties? Days: ___

Q28. Did you notice changes in your sense of taste? ☐ Not at all ☐ To some extent ☐ Significantly ☐ Yes Q29. On how many days after surgery did you experience taste disturbances? Days: ___

Q30. Did you notice changes in chewing food? ☐ Not at all ☐ To some extent ☐ Significantly ☐ Yes Q31. On how many days after surgery did you experience chewing difficulties? Days: ___ Q32. Did you experience difficulty opening your mouth? ☐ Not at all ☐ To some extent ☐ Significantly ☐ Yes Q33. On how many days after surgery did you experience incomplete mouth opening? Days: ___

4.4 Speech-Related Changes Q34. Did you notice changes in your voice? ☐ Not at all ☐ To some extent ☐ Significantly ☐ Yes Q35. On how many days after surgery did you experience speaking difficulties? Days: ___

Q36. Did you notice changes in your speech? ☐ Not at all ☐ To some extent ☐ Significantly ☐ Yes

Q37. Were your conversation partners able to understand you? ☐ Not at all ☐ To some extent ☐ Significantly ☐ Yes

4.5. Sleep Disturbances Q38. Did you experience difficulty falling asleep? ☐ Not at all ☐ To some extent ☐ Significantly ☐ Yes Q39. On how many days after surgery did you experience sleep disturbances? Days: ___

Q40. Did you experience sleep disturbances after surgery? ☐ Not at all ☐ To some extent ☐ Significantly ☐ Yes

Q41. Did you experience postoperative drowsiness? ☐ Not at all ☐ To some extent ☐ Significantly ☐ Yes

4.6. Appearance Q42. Did you notice changes in your appearance? ☐ Yes ☐ No Q43. Did the surgery and postoperative period proceed as expected for you? ☐ Yes ☐ No Q44. Was the course of surgery and postoperative recovery better than expected? ☐ Yes ☐ No Q45. Was the course of surgery and postoperative recovery worse than expected? ☐ Yes ☐ No

4.7. Quality of Life and Satisfaction Q46. Are you satisfied with the treatment you received? ☐ Yes ☐ No Q47. Would you recommend this treatment to others? ☐ Yes ☐ No Q48. Would you undergo the same treatment again if you experienced similar symptoms related to an impacted third molar? ☐ Yes ☐ No Q49. Do you feel that the problem causing your symptoms has been resolved? ☐ Yes ☐ No
